# Targeted knockout of the gene *OsHOL1* removes methyl iodide emissions from rice plants

**DOI:** 10.1038/s41598-021-95198-x

**Published:** 2021-08-23

**Authors:** Martina Carlessi, Lorenzo Mariotti, Francesca Giaume, Fabio Fornara, Pierdomenico Perata, Silvia Gonzali

**Affiliations:** 1 PlantLab, Institute of Life Sciences, Scuola Superiore Sant’Anna, Pisa, Italy; 2 Department of Agriculture, Food and Environment, University of Pisa, Pisa, Italy; 3 Department of Biosciences, University of Milan, Milan, Italy

**Keywords:** Plant physiology, Plant molecular biology, Natural hazards, Genetic engineering

## Abstract

Iodine deficiency represents a public health problem worldwide. To increase the amount of iodine in the diet, biofortification strategies of plants have been tried. They rely on the exogenous administration of iodine to increase its absorption and accumulation. However, iodine is not stable in plants and can be volatilized as methyl iodide through the action of specific methyltransferases encoded by the *HARMLESS TO OZONE LAYER* (*HOL*) genes. The release of methyl iodide in the atmosphere represents a threat for the environment due to its ozone depletion potential. Rice paddies are among the strongest producers of methyl iodide. Thus, the agronomic approach of iodine biofortification is not appropriate for this crop, leading to further increases of iodine emissions. In this work, we used the genome editing CRISPR/Cas9 technology to knockout the rice *HOL* genes and investigate their function. *OsHOL1* resulted a major player in methyl iodide production, since its knockout abolished the process. Moreover, its overexpression reinforced it. Conversely, knockout of *OsHOL2* did not produce effects. Our experiments helped elucidating the function of the rice *HOL* genes, providing tools to develop new rice varieties with reduced iodine emissions and thus more suitable for biofortification programs without further impacting on the environment.

## Introduction

Rice (*Oryza sativa* L.) is among the oldest cultivated plants and one of the most important cereal, still representing a staple food crop for about 2.5 billion people worldwide^1^. Its cultivation covers the 1% of the global land area^2^, and, with maize and wheat, it contributes to bring half of the calories consumed by the human population^3^. As a result, ingestion of rice may represent an important pathway for nutrients into human diet and biotechnologies have been developed to enrich rice varieties of essential vitamins and microelements^4^.

Iodine represents one of the most important micronutrients for human health, being necessary in the thyroid gland to synthesize the hormones triiodothyronine and thyroxine^5^. Iodine deficiency is one of the most serious public health problems worldwide, affecting almost one-third of the human population^6^. Usually cultivated crops and vegetables are poor sources of iodine. This is mainly due to the low content of the element in the soil, especially in rural areas distributed in mountainous and other inland zones^7^, where plants cannot find adequate amounts of the element^8^. The range of iodine in grain crops, for example, is estimated between 2 and 30 µg/kg, which is too low to meet the daily demand, being the iodine requirement for an adult around 150–200 µg per day^9^.

Iodine deficiency affects both developing and well-developed countries but is particularly severe in areas where rice represents the main staple food, being this species generally poor of iodine. In inland areas and in flooded fields, where rice is often cultivated, the iodine present in the soil is generally leached away, thus reducing its content in plants^10^. They, indeed, absorb this element mainly through the roots, being less than 0.2% the amount taken up by the atmosphere^11^. Moreover, polished rice contains only 0.055% of the element absorbed by the plant, and more than 70% is found in the straw^11^. A reduced phloematic transport is partially responsible of the low iodine amount in the seeds, but, remarkably, the process of iodine volatilization, that releases methyl iodide (CH_3_I) in the gaseous form, may also strongly contribute to it^8^. As a matter of fact, due to these emissions, the iodine foliar concentration in rice decreases exponentially with a half-life of 14 days^12^. Consequently, the element stored in the plant, potentially prone to be translocated to the edible grains, is drastically reduced.

Methyl halides, including CH_3_I, are produced from natural and anthropogenic activities and represent an important source of halogens in the atmosphere. Their breakdown products are involved in catalytic heterogeneous chemical reactions, which play an important role in the tropospheric chemistry and in the stratospheric ozone loss^13^. CH_3_I has a short lifetime compared to other methyl halides, and therefore a lower ozone depletion potential. However, iodine produced from methyl iodide can strongly influence the oxidizing capacity of the troposphere and induce a radiative effect affecting new particle formation^14^. The global production of CH_3_I is around 260–610 kt/year, and approximately 5% of this, about 16–29 kt/year, comes from rice paddies worldwide^15,16^: rice cultivation has therefore a major impact on the chemistry of the atmosphere. CH_3_I is mainly emitted during the early stages of rice growth, before maximum tillering, and particularly when rice is grown in soils with elevated halide concentrations^17^. Temperature is also important: a 1˚C temperature rise can increase the CH_3_I emission from rice by 10%^16^.

Methyl iodide emissions in plants involve enzymatic reactions catalyzed by halide methyltransferases (HMTs) or halide/thiol methyltransferases (HTMTs), which both have an S-adenosyl-L-methionine (SAM)-dependent methyl transferase activity and generate S-adenosyl-L-homocysteine as the by-product^18–20^. The identification of these enzymes in several higher plants^21^ indicates that their activity is widespread. However, the versatility shown for different substrates makes their physiological role still unclear. In an in vivo study performed in the model species *Arabidopsis thaliana*, Rhew et al*.*^22^ showed that methyl halides do not play a fundamental role in plant development. However, in soils with high salinity, like the salt marshes, the production of methyl halides can be considerably higher than in other ecosystems^23,24^. Thus, in halotolerant plants, methyl halides could be produced to maintain homeostatic levels of halide ions in the cytoplasm and to detoxify the plant from them, being their potential phytotoxicity widely demonstrated^8,25^. Moreover, in some groups of plants, such as the *Brassicaceae*, some HTMTs show a particular ability to methylate thiocyanate or bisulfide ions, produced from the hydrolysis of glucosinolates^20^. In such species, HTMTs could be involved in the detoxification of sulfur compounds^20,22^, and, in this case, halomethanes may simply be by-products of metabolism^26^.

Plant HMTs and HTMTs are encoded by small families of genes. The *HOL1* (*HARMLESS TO OZONE LAYER1*) gene, identified in Arabidopsis and thus named for the loss of function of the mutant enzyme*,* is the main responsible for methyl halide emissions in this species^22^. Other two homologous genes were identified in Arabidopsis, but their proteins seemed to play minor roles *in vivo*^27^. Homologous of these enzymes are widespread in the plant kingdom, including dicots, monocots, and unicellular algae^27^. In *Oryza sativa*, two genes were identified, *OsHOL1* and *OsHOL2*, which show, respectively, a 51% and a 57% similarity with Arabidopsis *HOL1*^28^. In in vitro studies, recombinant OsHOL proteins showed SAM-dependent methyltransferase activities towards halide and thiocyanate ions, with the highest effects exerted with iodide, suggesting a major involvement in the metabolism of iodine^28^.

Here, transgenic rice plants for the genes encoding the enzymes involved in iodine volatilization were produced to analyze the impact of each gene knockout, obtained by CRISPR/Cas9 technology, as well as of gene overexpression, on methyl iodide emissions. *OsHOL1* and the relative protein were identified as the main players involved in the process. To deepen our knowledge on the biochemical mechanisms underlining the methyl halides production, the subcellular localization of OsHOL1 and its ability to take part to multiprotein complexes were studied. We developed an effective mechanism to reduce methyl iodide emissions from rice plants and propose a possible strategy to enrich the iodine content of rice crops without further impacting on the environment.

## Results

### In rice plants, the halide methyl transferase genes are mainly expressed in leaves

The two genes of rice identified as homologous of *AtHOL1*^22^, *OsHOL1* and *OsHOL2*^28^, belong to different chromosomes. *OsHOL1* (*Os03g62670*) is located on chromosome 3 and organized into seven exons and six introns, which can be alternatively spliced in three predicted different isoforms (Fig. S1A). The longest full-length coding sequence (cds) (*Os03g62670.1*) corresponds to an open reading frame of 741 bp, coding for a 246 amino acid-long polypeptide. *OsHOL2* (*Os06g06040*) is located on chromosome 6 and organized into eight exons and seven introns, producing seven putatively different isoforms, with the longest full-length cds (*Os06g06040.1*) of 753 bp, encoding a protein of 250 amino acids (Fig. S1B).

The proteins OsHOL1 and OsHOL2 predicted from the two longest transcript isoforms are identified as “S-adenosylmethionine-dependent methyltransferases (SAM- or AdoMet-MTase), class I”, on the basis of their main conserved structural domains (Fig. S2A, B). They well aligned with other plant HMTs and HTMTs previously identified (Fig. S2C), even if showing some extra amino acid short sequences. OsHOL2 showed an 18 amino acid pre-sequence in the N-terminus, whereas OsHOL1 presented additional short stretches in the conserved central SAM-MTase domain, within the SAM binding site (Fig. S2C).

**Figure 1:**
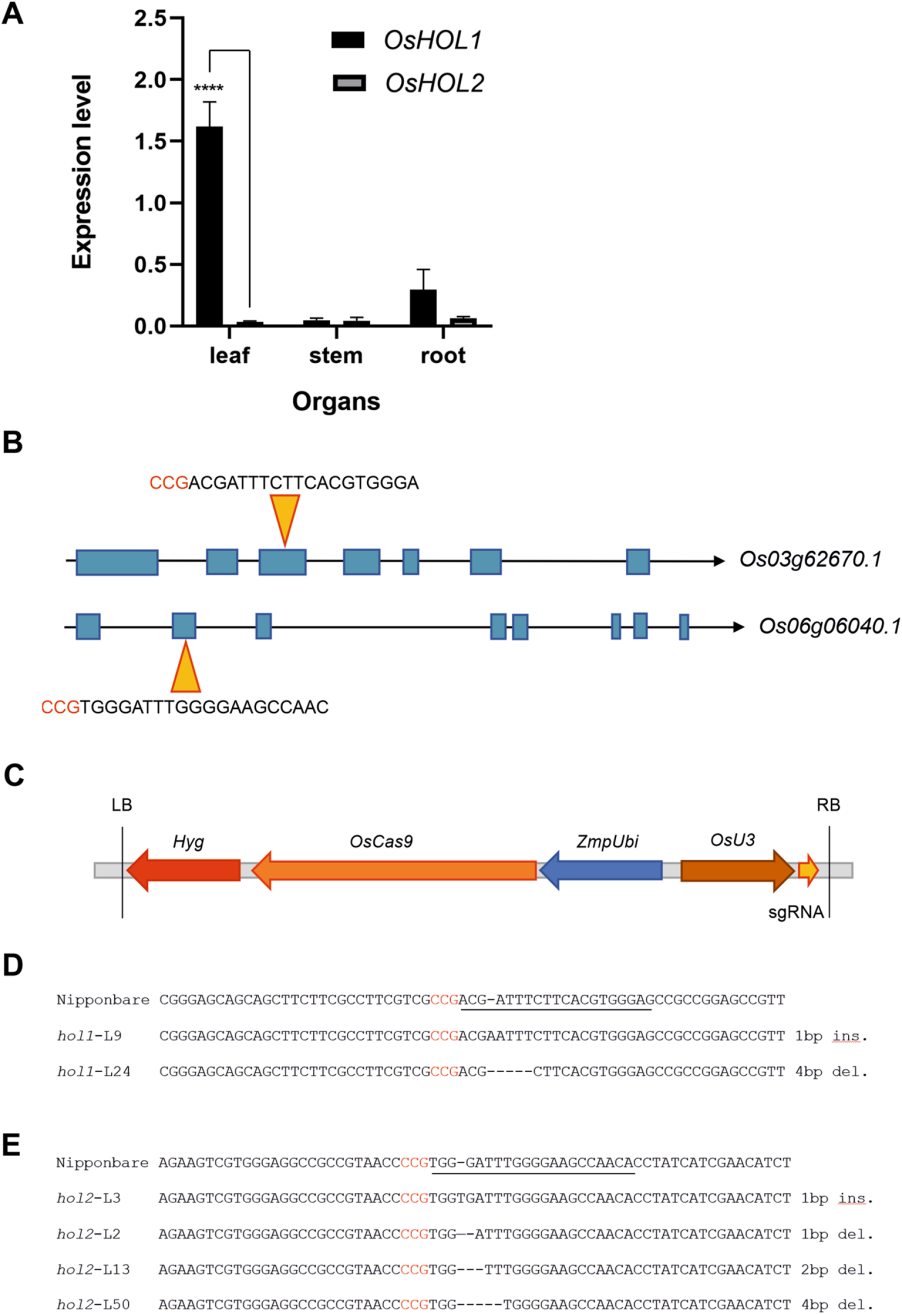
Expression of *OsHOL* genes in rice plants and CRISPR/Cas9 mutagenesis of the same genes. (**A**) RT-qPCR analysis of *OsHOL1* and *OsHOL2* in rice plant. Transcript levels were measured in leaves, stems and roots collected from 2-week-old plants. *OsHOL* expression levels were normalized to the reference gene *OsGAPDH*. Data points represent means ± s.d. of three biological replicates. T-test analysis was performed between *OsHOL1* and *OsHOL2* within each organ. Differences are indicated with asterisks. **** mean *P* ≤ 0.0001. (**B**) Schematic diagrams showing the exon–intron structures corresponding to the longest coding sequences of *OsHOL1* (*Os03g62670.1*) and *OsHOL2* (*Os06g06040.1*). Blue rectangles represent exons, connecting lines represent introns, yellow triangles indicate the position of the target sequence recognized by the gRNA produced for that gene, whose sequence is reported, with the protospacer adjacent motif (PAM) colored in red. (**C**) Schematic diagram of the T-DNA contained in the expression vector used in the study, the Ph-Ubi-CAS9-7-sgRNA vector. LB, left border; *Hyg*, hygromycin resistance gene; *OsCas9*, *Cas9* gene sequence optimized for *Oryza sativa*; *ZmpUbi*, *Zea mays Ubiquitin1* promoter; *OsU3*, *Oryza sativa U3* promoter; sgRNA, single-guide RNA sequence; RB, right border. Sequence of *OsHOL1* (**D**) and *OsHOL2* (**E**) around the gRNA target sequence (highlighted) in wild type Nipponbare plants and in CRISPR/Cas9 mutated lines generated in the study. The type of mutation is reported at the end of each edited sequence. In each sequence the PAM is colored in red.

To assess the expression levels of the two genes in rice under our experimental conditions, a quantitative real-time polymerase chain reaction (RT-qPCR) was performed analyzing leaves, stems, and roots sampled from young plants grown in soil. Transcripts of both genes were identified in all the organs analyzed, although at very different levels (Fig. 1A). *OsHOL1* appeared to be expressed more than *OsHOL2* and the difference was particularly high in leaves, where *OsHOL1* resulted mostly expressed. In roots and stems, both genes showed lower transcription rates.

### Mutagenesis of the halide methyl transferase genes by CRISPR/Cas9 technology

To mutagenize *OsHOL1* and *OsHOL2* through the CRISPR/Cas9 technology, candidate target sequences for specific gRNAs were identified and, according to the position along the genes and trying to minimize the likelihood of off-target effects, a gRNA targeting the third exon of *OsHOL1* and a gRNA targeting the second exon of *OsHOL2* were finally selected (Fig. 1B). Being the two genes characterized by multiple splicing isoforms (Fig. S1), the targeted exons were chosen among those present in all the predicted alternative transcripts. Each of the two gRNAs was assembled, cloned in a pOs-sgRNA entry plasmid, and then recombined with the Ph-Ubi-Cas9-7-sgRNA binary destination vector^29^.

Scutellum-derived rice calli were transformed with Agrobacterium cultures carrying the binary vectors with the CRISPR/Cas9-gRNA cassettes (Fig. 1C). Starting from a hundred rice seeds for each transformation, 54 and 24 in vitro regenerated plants were produced, respectively, for the mutagenesis of *OsHOL1* and *OsHOL2*. The presence of the T-DNA was attested in these plants by the PCR amplification of the hygromycin resistance gene contained in the genetic cassette (Fig. 1C, Fig. S3A). For each transgenic line, a 567 or 624 bp genomic fragment surrounding the gRNA target sequence in *OsHOL1* or *OsHOL2*, respectively, was then amplified (Fig. S3B) and sequenced. This allowed to distinguish between wild type and mutated copies of the genes, and homozygous, heterozygous and biallelic mutations, as well as chimeric mutations, were identified (Table S1).

For silencing of *OsHOL1*, 34 independent transgenic lines, with 18 lines bearing homozygous or biallelic mutations, 15 chimeras, and one showing a heterozygous mutation, were produced, whereas, for silencing of *OsHOL2* 20 independent mutated lines were obtained, of which 12 with homozygous or biallelic mutations, seven chimeras, and one with a heterozygous mutation (Table S1). Most of the mutations identified were single or very short insertions or deletions (indels) (1, 2 or 4 nucleotides) (Table S1, Fig. 1D,E), producing frameshift mutations. Some indels were larger (20 or 29 nucleotides) and could have led as well to frameshift mutations (Table S1). Others (e.g., 6 or 24 nt deletions), being in-frame, could have produced loss of a few amino acids in the relative polypeptide chains. The rest of the T_0_ regenerated transformed plants showed wild type alleles in *OsHOL1* and *OsHOL2*.

To exclude possible off-target effects, the gene *OsHOL2* was amplified and sequenced in some independent *OsHOL1* mutants. Indeed, considering the 8–10 bp seed region of the gRNA selected for *OsHOL1* mutagenesis, the only predictable off-target could have been in *OsHOL2*, which contains a sequence homologous to the gRNA target (Fig. S4A). However, as shown in Fig. S4B, no *OsHOL1* mutant line showed unintended mutations in the region of *OsHOL2* surrounding and downstream of the possible gRNA off-target. As far as the possible off-targets for the gRNA selected for *OsHOL2* mutagenesis, taking into the account the high specificity of its seed region we could exclude off-target effects in other rice genes, *OsHOL1* included.

The growth and the morphology of the regenerated T_0_ plants with or without mutations was similar. Apart from some common developmental responses to acclimatize from the stressful in vitro conditions, they did not show any peculiar vegetative phenotype and many of the *ex vitro* plantlets grew in soil till flowering. However, the number of spikelets resulted low and many developing seeds aborted, likely as a consequence of stress during in vitro culture.

### Knockouts in *OsHOL1* affected methyl iodide emissions from leaves

**Figure 2:**
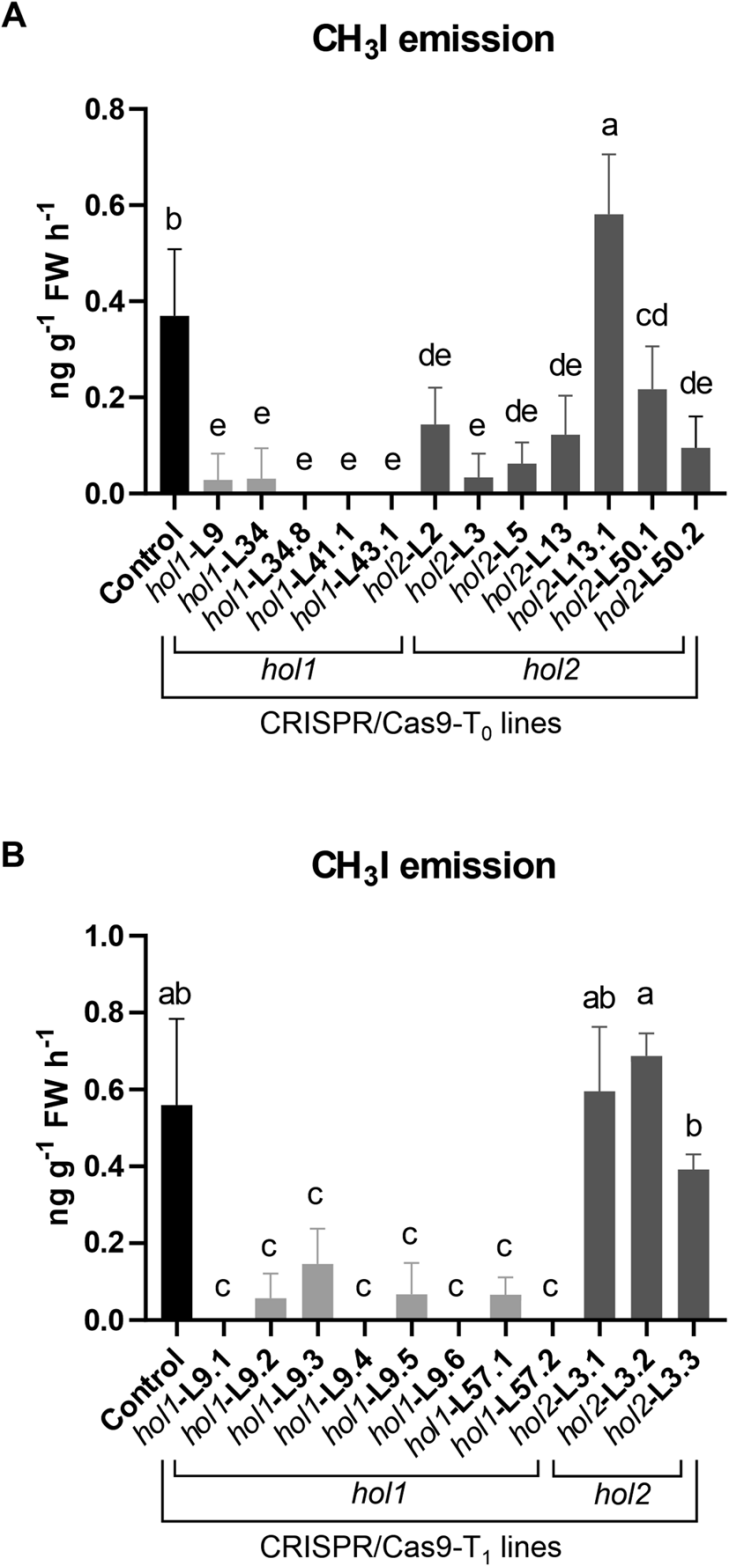
Methyl iodide emissions in CRISPR/Cas9 mutated lines. CH_3_I emissions were measured from T_0_ (**A**) and T_1_ (**B**) plants. Air samples from leaves were analyzed 24 h after incubation with 0.5 mM KI. Data points are the mean ± s.d. of four biological replicates for each line and two technical replicates per each biological sample. Data were subjected to one-way analysis of variance (ANOVA). Values followed by the same letter are not significantly different (Tukey’s HSD post-hoc test, *P* ≤ 0.05).

Single CRISPR/Cas9-T_0_ lines carrying different homozygous frameshift mutations in *OsHOL1* or *OsHOL2* were selected and the CH_3_I emissions from their leaves were measured by GC–MS. T_0_ regenerated plants with wild type alleles of the two genes were used as controls. All the lines mutagenized in *OsHOL1* showed very reduced emissions compared to control, whereas those mutagenized in *OsHOL2* presented variable phenotypes, some of them exhibiting emissions similar to control and others lower than control (Fig. 2A).

Plants belonging to T_1_ progenies produced by T_0_ lines carrying homozygous + 1 insertion mutations in *OsHOL1* or *OsHOL2* (lines *hol1*-L9 and *hol2*-L3, respectively) were grown and analyzed to check the presence of the mutations in the genes of interest. In both the T_1_ progenies, homozygous + 1 insertion mutations in *OsHOL1* or *OsHOL2* were confirmed in all the plants, whereas the *hol1*-L9.3 and *hol2*-L3.3 lines segregated for the hygromycin resistance gene, indicating the loss of the T-DNA genetic cassette (Fig. S5). Two T_1_ plants obtained from seeds collected from the heterozygous T_0_ *hol1*-L57 line (+ 1 bp/0 *OsHOL1* genotype) were also analyzed and found to contain homozygous + 1 insertion mutations in *OsHOL1* as well.

A characterization of CH_3_I emissions was carried out in all these T_1_ plants. Leaf samples from *hol1*-L9.1 ÷ L9.6 and *hol1*-L57.1-L57.2 plants, mutagenized in *OsHOL1*, showed absence or very reduced levels of CH_3_I emissions, while plants carrying mutations in *OsHOL2* (*hol2*-L3.1 ÷ L3.3) showed a phenotype not different from control (Fig. 2B).

Differently from T_0_ plants, all the CRISPR/Cas9 mutants belonging to the T_1_ generations could complete their life cycle: they flowered and developed spikelets and seeds. Due to the reduced number of T_1_ plants, a comparison with control Nipponbare plants in terms of grain yields or resistance to biotic or abiotic stresses could not be performed. However, the T_1_ mutant plants, either mutated in *OsHOL1* or *OsHOL2*, grew similarly to control and continued to show no visible peculiar phenotypes (Fig. S6).

### Overexpression of *OsHOL1* in rice plants and consequent methyl iodide emissions

A full-length cds of 741 nucleotides, corresponding to the predicted isoform *Os03g62670.1* of *OsHOL1* (Fig. S7), was amplified from rice leaves. Several independent transgenic lines overexpressing *OsHOL1* were produced after transformation of scutellum-derived rice calli with a genetic cassette expressing the cloned cds under the constitutive maize *Ubiquitin1* promoter. As in the case of the CRISPR/Cas9 mutagenized lines, all the regenerated plants showed no peculiar phenotype, excluding a reduced number of spikelets and seeds. This prompted us to ascribe the general reduced fertility shown by the transgenics to the in vitro regeneration process, rather to a genotype-specific effect.

**Figure 3:**
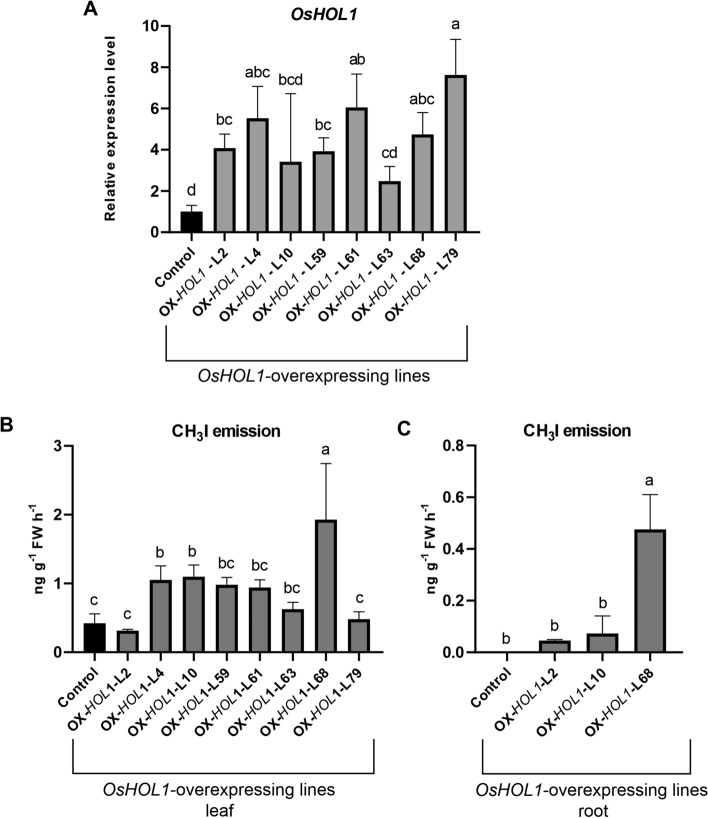
RT-qPCR and methyl iodide emission analyses in *OsHOL1* overexpressing lines. (**A**) Transcript levels of *OsHOL1* were measured in 2-week-old plants and expressed as relative values in comparison to the expression of control wild type plants. Values represent the means ± s.d. from three biological replicates. Data were subjected to one-way ANOVA. Values followed by the same letter are not significantly different (Tukey's post-hoc test, *P* ≤ 0.05). CH_3_I emissions measured from leaves (**B**) and roots (**C**) collected from *OsHOL1* overexpressing plants. Air samples from leaves or roots were analyzed 24 h after incubation with 0.5 mM KI. Data points are the mean ± s.d. of four biological replicates for each line and two technical replicates per each biological sample. Data were subjected to one-way ANOVA. Values followed by the same letter are not significantly different (Tukey’s HSD post-hoc test, *P* ≤ 0.05).

The level of overexpression of *OsHOL1* was checked by RT-qPCR in a subset of transgenics. Most of the lines showed an expression of the gene higher than control (Fig. 3A), and, when measuring their CH_3_I leaf-emissions, they also resulted, in many cases, higher than in wild type plants (Fig. 3B). Interestingly, whereas from roots sampled from wild type plants no emissions of CH_3_I were found, CH_3_I emissions were measured from roots isolated from *OsHOL1*-overexpressing lines, even if lower than those observed in leaves (Fig. 3C). This result was not unexpected being the ectopic expression of *OsHOL1* in the transgenic lines driven by a ubiquitous promoter.

### OsHOL1 is a dimeric protein with a complex subcellular localization

**Figure 4:**
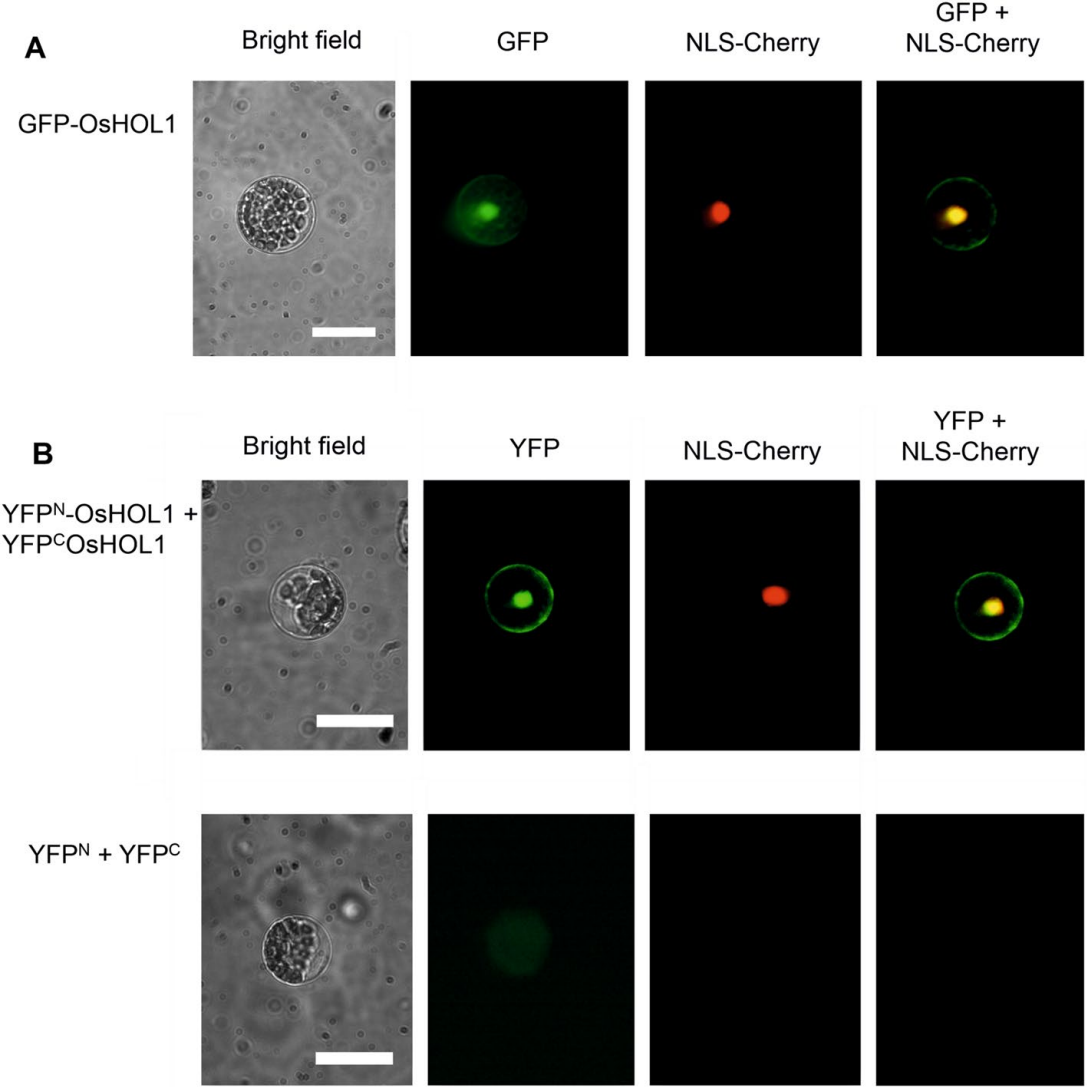
Cell localization and BiFC assay of OsHOL1 proteins. (**A**) Subcellular localization of OsHOL1, transiently expressed in Arabidopsis protoplasts as fusion proteins with GFP. GFP, NLS-Cherry RFP and merged GFP + NLS-Cherry RFP fluorescence and bright field images of protoplasts are shown. (**B**) Protein–protein BiFC interaction analysis of OsHOL1. OsHOL1-YFP^C^ + OsHOL1-YFP^N^ fusion proteins, and YFP^N^ + YFP^C^ split proteins were co-expressed in Arabidopsis protoplasts, as indicated in the picture. BiFC was detected from reconstitution of the YFP signal. Individual and merged images of YFP and NLS-Cherry RFP as well as bright field images of protoplasts are shown. Scale bars = 10 μm.

The GFP-OsHOL1 fusion protein, obtained from the expression of the cloned *Os03g62670.1* cds, was expressed in Arabidopsis protoplasts. A subcellular localization in both cytosol and nuclei was observed, and the superimposition in the nuclei of the GFP fluorescence with the red fluorescence of an NLS-Cherry-RFP construct was evident (Fig. 4A).

Being known the multimeric nature of some MTs, able to produce homodimer or homotetramer complexes^30^, protein–protein interaction studies were carried out. A Bimolecular Fluorescence Complementation assay clearly showed the formation of dimers of OsHOL1, as attested by the reconstitution of the fluorescence of the YFP protein thanks to the reassociation of the two split YFP fragments fused to two different OsHOL1 proteins (Fig. 4B). Notably, the YFP fluorescence exhibited the same subcellular localization of the GFP-OsHOL1 fusion protein (Fig. 4A), further substantiating the double subcellular localization of OsHOL1.

**Figure 5:**
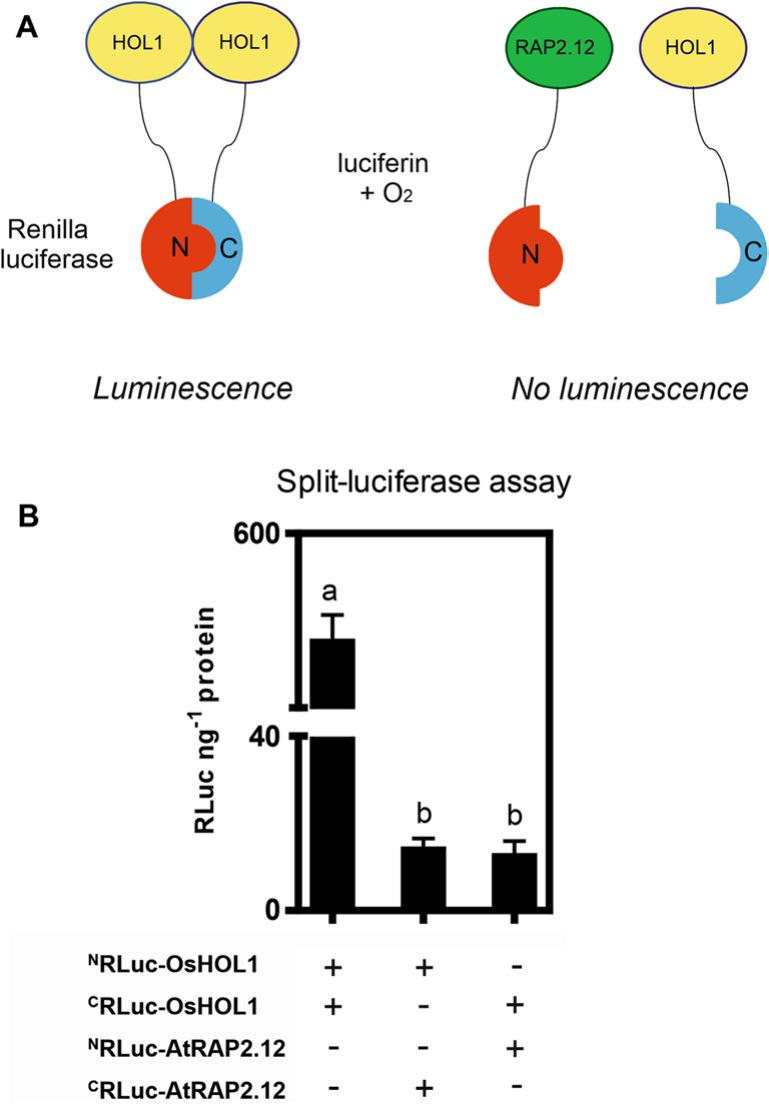
Split-Luciferase Complementation assay of OsHOL1 proteins. (**A**) Scheme of the Split-Luciferase Complementation assay designed to study the possible interactions between OsHOL1 and AtRAP2.12 proteins with the expected outcomes. (**B**) Quantitative result of the Split-Luciferase Complementation assay performed in Arabidopsis protoplasts through the expression of the fusion proteins NRLuc-OsHOL1, CRLuc-OsHOL1, NRLuc-AtRAP2.12, and CRLuc-AtRAP2.12. Different combinations of fusion proteins were analyzed, as reported in the picture. Data were expressed as luminescence units (RLuc) normalized to the protein content. One-way ANOVA followed by Tukey’s HSD post-hoc test (n = 4 biological replicates ± s.d.; *P* ≤ 0.05) was performed.

A Split-Luciferase Complementation assay also showed a strong interaction between the OsHOL1 partners of two *Renilla reniformis* luciferase split proteins, which were able to reconstitute a functional luciferase enzyme, again indicating the formation of homodimers of OsHOL1 (Fig. 5A, B). The specificity of the interaction between the two OsHOL1 proteins was confirmed by the lack of interaction of the same polypeptide with another protein, the Arabidopsis ERF-VII transcription factor RAP2.12, not functionally related with iodine metabolism but known to localize in cytosol and nuclei as well^31^ (Fig. 5B).

## Discussion

Iodine is traditionally not considered an essential element for higher plants, even if very recent studies have indicated its involvement in plant metabolism as a micronutrient^32^. Although several experiments showed how high concentrations of this element can be toxic for plants^8,33^, at low concentrations iodine can act as a growth enhancer, positively influencing growth and nutritional parameters^33–36^. It was also hypothesized that, by inducing an antioxidant response, low levels of iodine may protect plants from both biotic and abiotic stress conditions, such as salinity or heavy metals^8,37^. The impact of iodine on vegetables strongly depends on the species, the iodine form, the application method, and the properties of the substrate where plants are grown^36,37^. It is, however, undoubted that plants are able to take up iodine from the soil through the root system and from the air through the leaves, and that the quantity taken up is usually dependent on the amount of iodine available in the environment^8,37^.

Plants, however, do not only absorb iodine but also emit considerable amounts of it in the form of methyl iodide, thus reducing its content likely to avoid possible phytotoxic effects. However, in this way, they impact on the integrity of the stratosphere, being CH_3_I an ozone-depleting gas^13^. The CH_3_I volatilization process is mediated in plants by the *HOL* genes. There is only scattered evidence on possible tissue-specific expression patterns of these genes. The *TMT1 HOL* gene of *Brassica oleracea*, for example, showed high levels of expression in roots, stems and leaves^38^, and in Arabidopsis *AtHOL1* seemed to be ubiquitously expressed in the plant, including seedlings^27^. In *Raphanus sativus*, on the other hand, the activity of the HMT codified by *RsHOL* was found high in leaves but absent in roots^21^. Thus, it is still not clear whether plants can produce and volatilize methyl halides from all their tissues. In the present study, the expression of the two *HOL* genes of rice was analyzed in plants grown in soil without any exogenous iodine administration. Young plants were used, since previous data indicated that rice mostly volatilizes CH_3_I during the early stages of growth^13^. The RT-qPCR analysis we performed showed that the transcription levels of *OsHOL1* and *OsHOL2* were quite different, and that *OsHOL1* was the most expressed of the two genes, with a particularly high transcription rate in leaves. Our results agreed with the data reported in previous studies^28^, and also with those present in the Rice Expression Profile Database^39^, which show a sustained *OsHOL1* expression in leaves and a low transcription of *OsHOL2*, without preferential locations in the plant (Fig. S8). It seems therefore that shoots represent the main source of CH_3_I volatilization from rice plants.

To date, the HMTs of rice have not been fully characterized in vivo at the protein level. In our study, GFP-imaging studies indicated that GFP-tagged OsHOL1 proteins localized both in the cytosol and in the nuclei of plant cells. This represented an unexpected result and was not in accordance with previous hypothesis on a possible localization of these enzymes in the chloroplasts, where they may play a role in the detoxification of excess ions produced upon sulphate reduction^20^. More in accordance with our data, a study carried out in *Arabidopsis thaliana* and in *Brassica oleracea* detected HOL activity in the cytosol and not in the chloroplasts^40^. Based on our results, we can speculate that OsHOL1 proteins may mediate iodine detoxification processes in the cytoplasm as well as in the nuclei, where they may be necessary, for example, to prevent toxic actions of iodide on nucleic acids, or even their iodination^41,42^.

The HMTs of rice belong to the group of SAM-dependent MTs, enzymes implicated in a plethora of different essential cellular activities, including signal transduction, biosynthetic reactions, protein repair, chromatin regulation and gene silencing^30^. They are classified according to their structural folds in five classes. Most of the MTs currently known, including the two HMTs of rice, belong to the class I, the “Rossmann-fold” class^43^. Three-dimensional structures of some class I MTs revealed the presence of homodimeric or homotetrameric complexes, even if most of them should act as monomers^30^. We have shown that OsHOL1 was able to interact with itself in vitro and this suggests that it can form homodimers in vivo. As far as we know, this is the first evidence indicating a possible dimeric structure of an HMT in plants, since previous studies characterized as monomeric other HMTs, included AtHOL1^19,44,45^. The formation of homodimers was highlighted in other classes of plants MTs, for example in some of the type-1 family of SAM-dependent *O*-methyltransferases^46^. These enzymes are characterized by an auxiliary N-terminal domain that is required for the homodimerization and their structure consists of two monomers self-associating to form a homodimer, in which the central core is represented by the two dimerization domains, with the dimer interface contributing to the substrate binding, while the SAM binding domains are located externally^47^. Further studies are necessary to confirm the tridimensional structure of OsHOL1 in vivo, its possible dimeric nature, and, eventually, the specific domains involved in the dimerization. However, based on what already known in other MTs, dimerization could be of functional importance for the catalytic activity of the enzyme and its substrate discrimination.

In this study, we confirmed how the CRISPR/Cas9 system was an effective tool to mutagenize rice genes, thus representing a powerful tool which can contribute to the genetic improvement of this crop. The two major limits of this genome editing technology are the possible unfavorable editing at other sites in the genome^48^ and the on-target efficiency^49^. However, also possible on-target unexpected effects, such as large deletions, inversions, and insertions, were recently described^50^. The results we obtained showed the efficiency of both the selected gRNAs and the CRISPR/Cas9 vectors used. Gene knockouts were successfully generated in both *OsHOL1* and *OsHOL2* from T_0_ plants, and, in many cases, they consisted in single or very short indels inducing homozygous frameshift mutations. We did not extensively analyze off-target effects in the T_0_ edited plants, except for the predictable one in *OsHOL2* sequence in CRISPR/Cas9 *OsHOL1* mutants. This was excluded in three independent lines. The relevant number of independent T_0_ mutants produced for each gene, all showing phenotypes very similar among them and to wild type controls, did not suggest the existence of additional mutations. However, in the lines which will be finally maintained as reference stable knockout mutants for the two genes of interest, it would be appropriate to perform an in-depth whole-genome sequencing to definitively exclude such effects.

In our study a significant percentage of T_0_ plants resulted homozygous or biallelic for the mutated genes. As expected for the Cas9 enzyme and as already observed in rice plants, most of these mutations consisted of short indels (1 ~ 6 bp) in the close proximity of the protospacer motif, confirming that this kind of mutations frequently occur at an early stage of T_0_ rice plant development^51^. Single heterozygous mutations were also identified. This genotype, with the compresence of a mutated and a wild type allele, can be observed when mutations affect only one copy of the target gene in the embryogenic cell. However, it is also possible that mutations occurred after the division of the transformed embryogenic cell: in this case, the plant regenerated may be chimeric^51^. In such occurrence, heterozygote genotypes can also derive from chimeric tissues where some cells are homozygous for the mutation and others maintain the wild type genome. Many chimeras were produced in this study and identified in the regenerated T_0_ plants, confirming previous reports for CRISPR/Cas9-edited rice plants^51^. Based on the length of the indels obtained, most of which producing frameshifts, we could conclude that many homozygous mutations generated in T_0_ plants led to a loss of function of the genes of interest.

To determine if CRISPR/Cas9-mediated genome editing in *OsHOL1* and *OsHOL2* was heritable, we analyzed the T_1_ generations deriving from single T_0_ plants. First studies carried out in *Arabidopsis thaliana* reported that many mutations created by the CRISPR/Cas9 system occur in somatic cells and thus only a limited number of edited plants inherits the mutation in the subsequent generations^52^. In our study, inheritance of the homozygous mutations was observed in all T_1_ populations studied, suggesting that the CRISPR/Cas9 targeted modifications had occurred in single embryogenic cells. Moreover, all T_1_ plants deriving from T_0_ homozygous lines resulted homozygous for the same mutation identified in the progenitor plant, indicating that these mutations were stable and transmissible to the next generation, in accordance with the Mendelian laws of inheritance. Therefore, from these first analyses, it can be concluded that the mutations obtained in *OsHOL1* and *OsHOL2* T_0_ lines occurred in the germline cells and were inherited by T_1_ plants. Furthermore, in both groups of CRISPR/Cas9-edited plants, segregant T_1_ individuals, which lost the exogenous genetic cassette while maintaining the mutation in the gene of interest, were identified. Further analyses will be however necessary to ascertain the stability and heritability of these mutations in the following generations.

The most intriguing part of the study was the analysis of the effects of *OsHOL1* silencing or overexpression on methyl halide production *in planta*. Methyl iodide emissions were reported in different plant species^13,15,21^ and previous studies focused on methyl iodide quantification in open fields^13,15^ or in vitro systems^40^. We used an experimental set-up that allowed quantification of emissions from single plants. Remarkably, the *OsHOL1* homozygous knockout lines showed a significant reduction in CH_3_I emissions from their leaves, which, in some cases, were completely abolished. This phenotype was observed in all the different T_0_ lines analyzed and was also inherited from all the T_1_ plants bearing the same mutations. In the case of *OsHOL2*, the emission levels appeared variable among the different T_0_ homozygous mutant lines, but never eliminated, whereas in the T_1_ lines the CH_3_I production was identical to control. Since T_0_ plants, differently from their offspring, appeared somehow stressed as a consequence of the in vitro regeneration process, we assumed that comparison between control and mutant lines was more reliable when T_1_ plants were analyzed. Based on that, we concluded that the knockout of *OsHOL2* did not have a significant impact on the volatilization of methyl iodide from rice leaves.

As a natural completion of the study, transgenic lines overexpressing *OsHOL1* were produced and many of them showed higher CH_3_I emissions from leaves compared to control. Furthermore, in some lines methyl iodide emissions were also measured from roots, differently from wild type roots, where CH_3_I levels appeared negligible. These results confirmed the positive correlation between *OsHOL1* gene expression and methyl halide production.

The higher expression levels and the lower emissions measured when mutagenized, as well as the higher emissions measured when overexpressed, make of *OsHOL1* the main player in the iodine volatilization from rice. Our data did not support for the moment a possible active role of OsHOL2 in the methyl iodide production, at least in leaves. On the other hand, the absence of CH_3_I emission from wild type roots makes not plausible to hypothesize a possible activity of OsHOL2 in these organs as well. However, since the HMT activity of OsHOL2 was measured *in vitro*^28^, it is still possible that this enzyme carries out its action in other tissues of the plants or in specific developmental stages.

All the results here obtained were consistent with previous analyses performed in Arabidopsis, in which *Athol1* mutants showed dramatically lower methyl halide emissions compared to wild type plants, in spite of the existence of other two *HOL* genes, which thus appeared to play only marginal roles *in vivo*^22,27,53^. Furthermore, exogenous iodine administrations resulted able to increase *AtHOL1* transcription^53^. If this is also the case of rice plants, a possible increase of *OsHOL1* transcription, for example as a consequence of higher iodine availability in the environment, would lead to a further increase in CH_3_I emissions from leaves, similarly to what observed in the *OsHOL1* overexpressing lines. As a result, iodine-biofortification programs based on the use of exogenous iodine may lead to a further increase in methyl iodide production from rice crops, and therefore to an even more severe threat to the atmosphere.

The strong depressing effect of *OsHOL1* knockout on CH_3_I emissions represents an important achievement in the perspective of a genetic improvement of this crop through the production of new varieties less hazardous towards the chemistry of the atmosphere. At the same time, such varieties would be ideal candidates for the establishment of iodine-biofortification programs. As a matter of fact, since silencing of *AtHOL1* resulted in a significant increase of iodine content in Arabidopsis plants^53^, it is as much plausible that *OsHOL1* silencing may lead to increase of iodine in rice plants. The development of new rice varieties with low OsHOL1 activity, provided this does not affect their agronomic performance, will therefore represent a necessary and unavoidable strategy to effectively enrich of iodine rice plants, possibly increasing its translocation to the seeds and thus making this micronutrient more available for the human diet, without having more negative impact on the environment.

## Methods

### Plant material

Seeds of *Oryza sativa*, ssp. Japonica, cv. Nipponbare, from the Genetic Stocks Oryza (GSOR) Collection (Dale Bumpers National Rice Research Center, AR, USA), were used. Seeds were sterilized with 70% ethanol for 1 min followed by 30 min in 6% sodium hypochlorite with shaking and washed six times for 15 min in sterile water. To examine the expression of *HOL* genes, rice seeds were pre-germinated in water in the dark for 3 days and then sown in soil, placing three/four seeds in each pot. Plants were grown in a growth chamber at 28 °C day/24 °C night, with a 12 h photoperiod, 150/200 μmol photons m^−2^ s^−1^ and 35% relative humidity. Roots, shoots and single leaves were collected after 2 weeks and stored at − 80 °C. For callus induction, 100 sterilized seeds, 10 per Petri dish, were inoculated on modified callus induction media supplemented with vitamins and 2,4-Dichlorophenoxyacetic acid (2,4-D) and incubated in darkness at 28 °C for 2 weeks^54^. Seeds of *Arabidopsis thaliana*, Columbia-0 ecotype, were used. For protoplast isolation, seeds were sown in a hydroponic system^55^ and plants were cultured in a growth chamber for 3 weeks at 23 °C/19 °C night with a 12 h photoperiod at 120 μmol photons m^–2^ s^–1^.

### Expression analysis of OsHOL1 and OsHOL2 genes

Total RNA was extracted from rice leaves and roots as previously described^56^. Quality and integrity of RNA were checked by electrophoresis analysis on 1% agarose gel. RNA concentration was measured with a Multiskan Microplate Spectrophotometer (Thermo Fisher Scientific, USA). RNA was reverse transcribed using the “iScript TM cDNA synthesis kit” (Bio-Rad Laboratories, USA). RT-qPCR analysis was performed with technical duplicates in an ABI Prism 7300 Sequence Detection System (Thermo Fisher Scientific), using the “PowerUP SYBR Green Mastermix” (Thermo Fisher Scientific). Three biological replicates were analyzed**.** The reference gene *OsGAPDH* and the target genes *OsHOL1* and *OsHOL2* were amplified using the oligonucleotide primers listed in Table S2. Relative gene expression was calculated using the geometric averaging method^57^.

#### Sequence analyses

DNA and protein sequences were analyzed through online-available sequence analysis software. Multiple Sequence Alignments were carried out by using the CLUSTALW (Version 1.83) tool (www.genome.jp/tools-bin/clustalw). Protein Domains and Macromolecular Structures analyses were performed through the tools available on the NCBI website (www.ncbi.nlm.nih.gov/Structure/index.shtml). Sanger sequencing of PCR products were performed at Eurofins Genomics (Eurofins Scientific, Germany).

#### Targeted mutagenesis of OsHOL1 and OsHOL2 genes using the CRISPR-Cas9 system

The sequences of the genes *Os03g62670* (*OsHOL1*) and *Os06g06040* (*OsHOL2*) were downloaded from the Rice Genome Annotation Project website (http://rice.plantbiology.msu.edu/). Suitable DNA target sequences for guide RNA (gRNA) design were selected using the “CRISPR-P 2.0 design tool” (http://crispr.hzau.edu.cn/CRISPR2/). The gRNAs were synthesized, annealed, and inserted at the BsaI sites in the pOs-sgRNA entry vector^29^. Binary T-DNA pH-Ubi-Cas9-7 vectors^29^ for co-expression of *Cas9* and the selected gRNAs were produced through Gateway™ recombination technology using the “LR Clonase II™” enzyme mix (Thermo Fisher Scientific). The sequence of each gRNA cassette after recombination in the expression vector was analyzed by PCR, using the primers listed in Table S2.

#### Generation of OsHOL1 and OsHOL2 knock-out plants and analysis of mutations

The expression vectors containing the CRISPR/Cas9 genetic cassettes containing the gRNAs targeted the genes of interest were introduced into *Agrobacterium tumefaciens* strain EHA105 by the freeze–thaw method. Rice transformation was performed starting from embryogenic calli according to the protocol described by^58^, as modified by^54^. Genomic DNA was extracted from T_0_ transgenic plants grown in soil using the “CTAB extraction method” and amplified for the hygromycin resistance gene using the primers listed in Table S2. Genomic fragments of 567 or 624 bp containing the target site were amplified from the genomic DNA of all the hygromycin PCR-positive plants using the specific *OsHOL1* and *OsHOL2* primers listed in Table S2. The PCR products were purified using the “Wizard® Genomic DNA Purification Kit” (Promega, USA) and then sequenced using the same PCR primers. The relative chromatograms were analyzed by Synthego “ICE CRISPR analysis tool” (https://ice.synthego.com/#/).

#### Vector construction and production of OsHOL1 overexpressing lines

The expressed cds of *OsHOL1* was amplified from total RNA extracted from leaf tissue. First-strand cDNA was synthesized from RNA using the “Superscript IV® reverse transcriptase” (Thermo Fisher Scientific), according to the manufacturer’s instructions, and then amplified by PCR using the “Phusion® High-Fidelity DNA Polymerase” enzyme (Thermo Fisher Scientific) and the primers listed in Table S2. The purified full-length cds was cloned into the pENTR™/D-TOPO vectors (Thermo Fisher Scientific) and then recombined with the pIPKb003 destination vector^59^ using the “LR Clonase II™” enzyme mix (Thermo Fisher Scientific). The expression vector was mobilized into Agrobacterium strain EHA105 for transformation of rice calli and transgenic plants were produced as above described. Total RNA was extracted from leaf tissues of T_0_ plants using the “TRIzol™ Reagent” (Sigma-Aldrich, Merck, Germany) and the expression level of *OsHOL1* was measured by RT-qPCR, as above described.

#### Measurement of methyl iodide emissions

Rice leaves (0.2 g per sample) were collected from control and transgenic plants, cut in small pieces, and incubated in a 22-ml sealed vial containing 0.5X Murashige & Skoog (MS) solution and 0.5 mM potassium iodide (KI) for 24 h at 23 °C under gentle shaking. Methyl iodide emissions were measured using a gas chromatography-tandem mass spectrometry (GC–MS/MS) CP-3800 gas chromatograph coupled to Saturn 2200 quadrupole ion trap mass spectrometer (Varian Analytical Instruments, Walnut Creek, CA, USA). 1 mL of the head space gas from the sealed vials was injected into a Mega PS264 capillary column (30 m × 0.25 mm i.d., 3.00 μm film thickness) (Mega, Milano, Italy). The carrier gas was helium, which was dried and air free, with a linear speed of 60 cm s^−1^. The oven temperature was maintained at 80 °C for 5 min. Injector and transfer line were set at 150 °C and the ion source temperature at 200 °C. Full scan mass spectra were obtained in EI + mode with an emission current of 10 µA and an axial modulation of 4 V. Data acquisitions was from 10 to 250 Da at a speed of 1.4 scan s^−1^. CH_3_I was identified by comparison with the retention time and spectrum ions (m/z) of a methyl iodide standard (Merck, Germany). The quantification was performed using the calibration curve with this compound. The minimum level of quantification (LOQ) and the minimum level of detection (LOD) were monitored daily with standard and with the signal/noise ratio, respectively. The same experimental setup was used to quantify methyl iodide emissions produced by rice roots (0.55 g per sample).

#### Subcellular localization studies in Arabidopsis thaliana protoplasts

The entry vector containing the cds of *OsHOL1* was recombined with the p2FGW7 destination vector^60^ using the Gateway recombination protocol, as previously described, to produce a *35S*:*GFP-OsHOL1* expression plasmid. Arabidopsis mesophyll protoplasts were isolated and transfected with 5 μg of plasmid, as described in^61^. An mCherry-NLS–RFP-containing vector^62^ was used to visualize cell nuclei. For GFP and RFP imaging, Arabidopsis protoplasts were analyzed using a Leica THUNDER Imager Model Organism fluorescence microscope (Leica Microsystems, Germany).

#### Study of protein–protein interactions through Split-Luciferase Complementation assay

*OsHOL1* cds, from the entry vector, was recombined into the Gateway-compatible split-luciferase system composed of pDuExAn6 and pDuExDn6 vectors^63^. The same vectors containing *AtRAP2.12*^31^ were gently provided by Dr. Alicja Kunkowska, Institute of Life Sciences, Scuola Superiore Sant'Anna, Pisa (Italy). Arabidopsis protoplasts, isolated as above described, were transfected with 5 μg of each plasmid. After an overnight incubation, protoplasts were pelleted and sea pansy (*Renilla reniformis*) luciferase activity was measured using the “Luciferase Reporter Assay System” (Promega). Luminescence intensity, measured with a Lumat LB 9507 Tube Luminometer (Berthold Technologies GmbH & Co., Germany), was normalized to the protein content of each sample determined through the Bradford protein assay (Bio-Rad).

#### Study of protein–protein interactions through Bimolecular Fluorescence Complementation (BiFC) assay

Vectors for the expression of OsHOL1 proteins fused to the N-terminal or C-terminal YFP fragments were generated by Gateway recombination of the *OsHOL1* cds entry vector with the destination vectors pE-SPYNE-GW and pE-SPYCE-GW^64,65^. Arabidopsis protoplasts, isolated as above described, were transformed with 5 μg of each BiFC vector. An mCherry-NLS–RFP-containing vector^62^ was used to visualize the cell nuclei. YFP and RFP fluorescence was visualized using a Leica THUNDER Imager Model Organism fluorescence microscope (Leica Microsystems).

## Supplementary Information

Supplementary Information.
