# Supplementary material for: Targeted knockout of the gene OsHOL1 removes methyl iodide emissions from rice plants
Source: Sci Rep. 2021 Aug 23;11:17010. doi: 10.1038/s41598-021-95198-x (PMC8382704; doi:10.1038/s41598-021-95198-x)
Supplement: Supplementary file 1 — Supplementary Information. [file 41598_2021_95198_MOESM1_ESM.pdf]

# Targeted knockout of the gene *OsHOL1* removes methyl iodide emissions from rice plants

Martina Carlessi[1], Lorenzo Mariotti[2], Francesca Giaume[3], Fabio Fornara[3], Pierdomenico Perata[1], Silvia Gonzali[1]

[1]PlantLab, Institute of Life Sciences, Scuola Superiore Sant'Anna, Pisa, Italy

[2]Department of Agriculture, Food and Environment, University of Pisa, Pisa, Italy

[3]Department of Biosciences, University of Milan, Milan, Italy

## Supplementary information

A

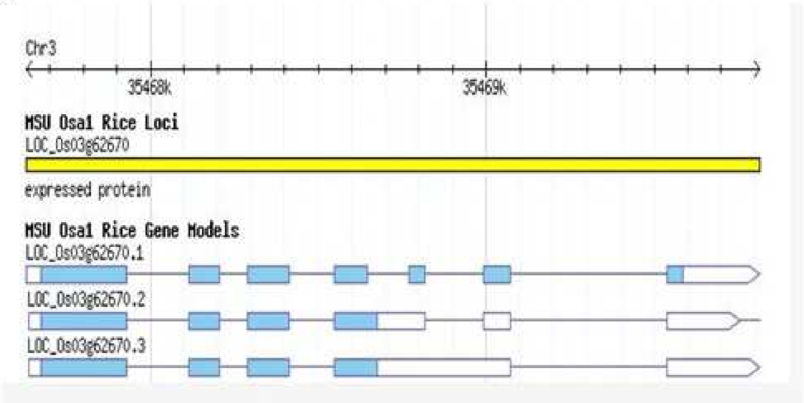


B

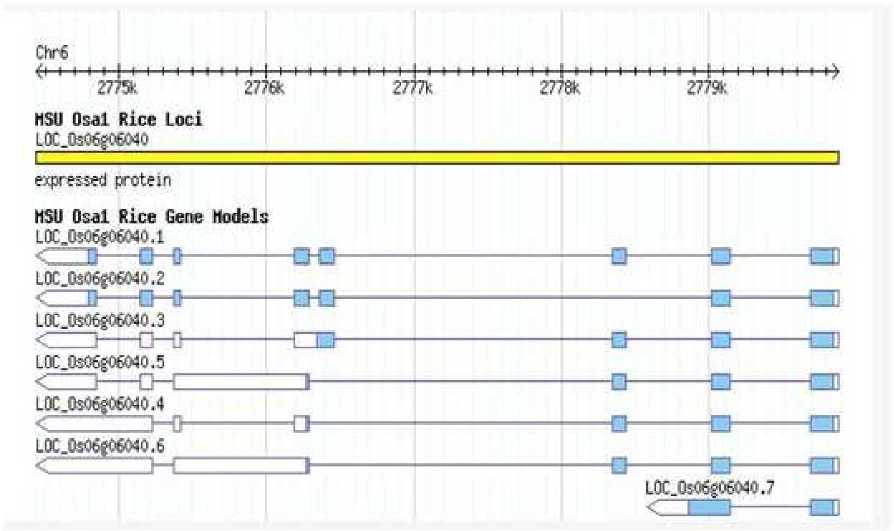


**Figure S1.** Gene structure of *OsHOL1* and *OsHOL2*. Exon-intron structure and predicted isoforms of *OsHOL1* (*LOC_Os03g62670*) (**A**) and *OsHOL2* (*LOC_Os06g06040*) (**B**) genes and relative positions on chromosomes 3 and 6, respectively, are shown. The images were downloaded from the Rice Genome Annotation Project Database website (http://rice.plantbiology.msu.edu/).

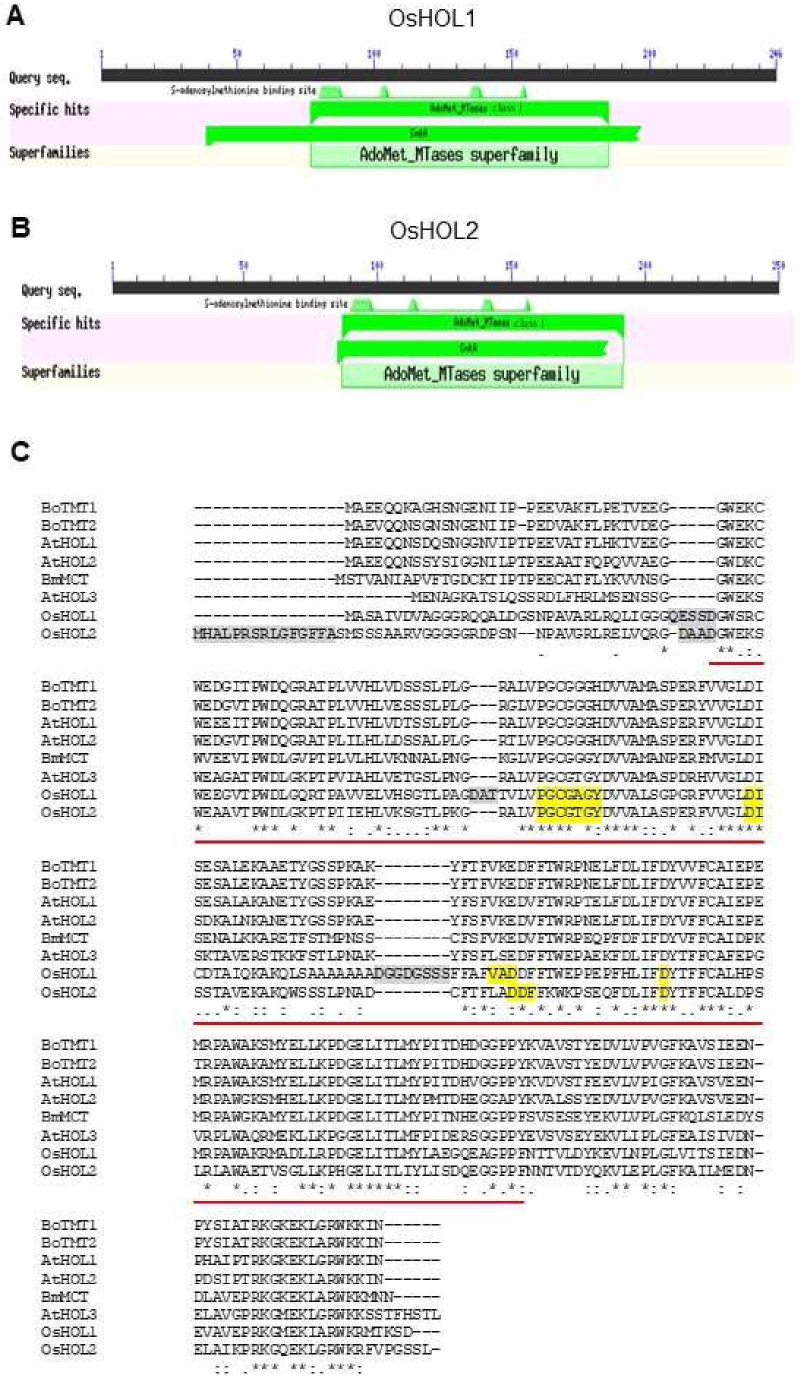


**Figure S2.** Characterization of OsHOL1 and OsHOL2 proteins. Protein Domains and Macromolecular Structures analyses of OsHOL1 (**A**) and OsHOL2 (**B**) were performed through the tools available on the NCBI website (www.ncbi.nlm.nih.gov/Structure/index.shtml). The conserved amino acid domains are indicated under the sequence. (**C**) ClustalW alignment (https://www.genome.jp/tools-bin/clustalw) of OsHOL1 and OsHOL2 with other TMTs and HTMTs identified in higher plants. The SAM-dependent MT domain is highlighted in red. The SAM-binding domains in OsHOL1 and OsHOL2 are highlighted in yellow. The sequences of OsHOL1 and OsHOL2 not aligning with the other proteins are highlighted in grey. '*' indicates positions which have a single, fully conserved residue. ':' indicates that one of the 'strong' groups is fully conserved. '.' indicates that one of the 'weaker' groups is fully conserved. CLUSTAL W Version 1.83 was used.

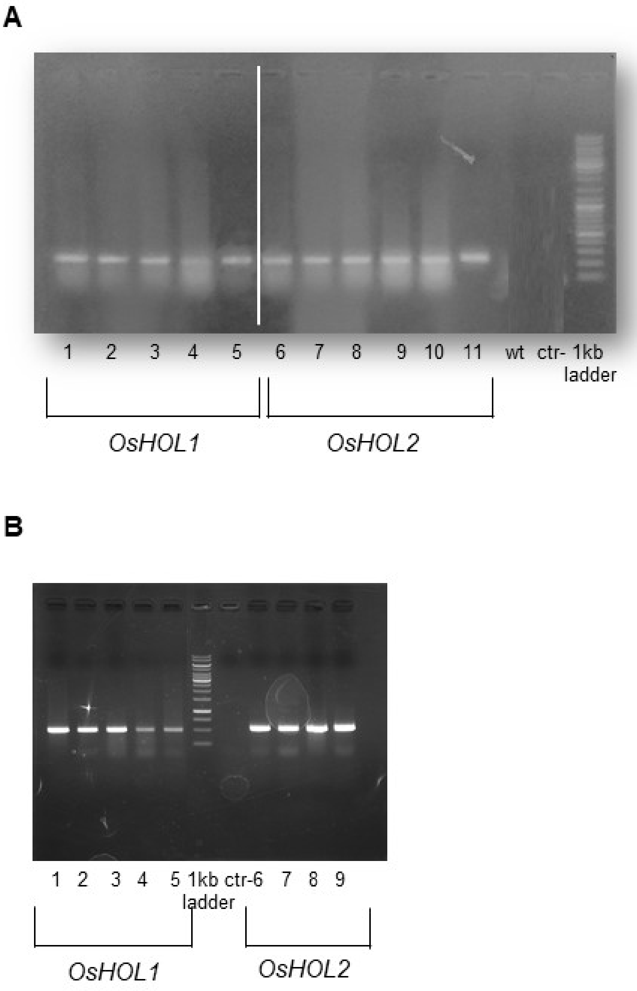


**Figure S3.** PCR analysis of $T_0$ CRISPR/Cas9 mutagenized lines. PCR amplification for the hygromycin resistance gene (**A**) and for the genomic region surrounding the gRNA target sequence in the gene of interest (**B**). Wild type (wt) sample (untransformed plant) and Ctr- sample (PCR negative control) were included in the analyses. Only the results of a subset of $T_0$ plants carrying the construct for the mutagenesis of *OsHOL1* or *OsHOL2* are shown. Lane positions in the gels and corresponding lines are as follows: *OsHOL1*: 1=L1.1, 2=L1.2, 3=L1.3, 4=L1.5, 5=L1.10; *OsHOL2*: 6=L2, 7=L3, 8=L5, 9=L11.3, 10=L11.8, 11=L11.2. The GeneRuler$^{TM}$ 1kb DNA ladders (Thermo Scientific) are shown in the gels. Original full-length gels are presented in Figure S9.

**A**

```
CLUSTAL 2.1 multiple sequence alignment


OsHOL1          ACACGGCCATCCAGAAGGCGAAGCAGCTGTCGGCGGCGGCGGCGGCGGCGGCTGACGGCG
OsHOL2          GTACAGCTGTGGAGAAGGCTAAGCAGTGGTCATCATCTTTGCCAAATGCAG---------
                  ** **  *  ******* ******  ***  *  *   * *    ** *

OsHOL1          GCGACGGGAGCAGCAGCTTCTTCGCCTTCGTCGCCGACGATTTCTTCACGTGGGAGCCGC
OsHOL2          ---------------ACTGTTTTACTTTTCTGGCTGACGATTTCTTCAAGTGGAAACCAA
                                **  **  * **  * ** ************* **** * **

OsHOL1          CGGAGCCGTTCCATCTCATCTTCGACTACACATTCTTCTGTGCTCTGCATCCGTCGATGA
OsHOL2          GTGAACAATTTGATCTTATTTTCGATTATACGTTCTTTTGTGCACTTGATCCAAGCTTGA
                  ** *  **  **** ** ***** ** ** ***** ***** **  ****     ***
```

**B**

```
CLUSTAL 2.1 multiple sequence alignment


Nipponbare          TGCAGACTGTTTTACTTTTCTGGCTGACGATTTCTTCAAGTGGAAACCAAGTGAACAATT
hol1_L34.8          TGCAGACTGTTTTACTTTTCTGGCTGACGATTTCTTCAAGTGGAAACCAAGTGAACAATT
hol1_L41.3          TGCAGACTGTTTTACTTTTCTGGCTGACGATTTCTTCAAGTGGAAACCAAGTGAACAATT
hol1_L57            TGCAGACTGTTTTACTTTTCTGGCTGACGATTTCTTCAAGTGGAAACCAAGTGAACAATT
                    ************************************************************

Nipponbare          TGATCTTATTTTCGATTATACGTATGTGCTAATTTTATCCTGTTGATTTCGTATTCTTGT
hol1_L34.8          TGATCTTATTTTCGATTATACGTATGTGCTAATTTTATCCTGTTGATTTCGTATTCTTGT
hol1_L41.3          TGATCTTATTTTCGATTATACGTATGTGCTAATTTTATCCTGTTGATTTCGTATTCTTGT
hol1_L57            TGATCTTATTTTCGATTATACGTATGTGCTAATTTTATCCTGTTGATTTCGTATTCTTGT
                    ************************************************************

Nipponbare          GGCATCTTAAATGACCTTTATTAATATACACAGGTTCTTTTGTGCACTTGATCCAAGCTT
hol1_L34.8          GGCATCTTAAATGACCTTTATTAATATACACAGGTTCTTTTGTGCACTTGATCCAAGCTT
hol1_L41.3          GGCATCTTAAATGACCTTTATTAATATACACAGGTTCTTTTGTGCACTTGATCCAAGCTT
hol1_L57            GGCATCTTAAATGACCTTTATTAATATACACAGGTTCTTTTGTGCACTTGATCCAAGCTT
                    ************************************************************

Nipponbare          GAGGTTGGCTTGGGCAGAAACAGTTAGTGGGCTTCTAAAACCTCATGGAGAGCTAATCAC
hol1_L34.8          GAGGTTGGCTTGGGCAGAAACAGTTAGTGGGCTTCTAAAACCTCATGGAGAGCTAATCAC
hol1_L41.3          GAGGTTGGCTTGGGCAGAAACAGTTAGTGGGCTTCTAAAACCTCATGGAGAGCTAATCAC
hol1_L57            GAGGTTGGCTTGGGCAGAAACAGTTAGTGGGCTTCTAAAACCTCATGGAGAGCTAATCAC
                    ************************************************************

Nipponbare          CCTTATATATTTGGTAACTGAAGAATCTATCTATTCTTTTGTTTACTTTTCCATTGAGGA
hol1_L34.8          CCTTATATATTTGGTAACTGAAGAATCTATCTATTCTTTTGTTTACTTTTCCATTGAGGA
hol1_L41.3          CCTTATATATTTGGTAACTGAAGAATCTATCTATTCTTTTGTTTACTTTTCCATTGAGGA
hol1_L57            CCTTATATATTTGGTAACTGAAGAATCTATCTATTCTTTTGTTTACTTTTCCATTGAGGA
                    ************************************************************
```

**Figure S4.** ClustalW alignment (https://www.genome.jp/tools-bin/clustalw) of (**A**) the region of *OsHOL1* containing the gRNA target sequence (green) close to the PAM sequence (red) and of the homologous region of *OsHOL2* with the possible off-target (light blue) close to the weak PAM sequence (yellow), and (**B**) of the region of *OsHOL2*, including the possible off-target sequence (highlighted in yellow), amplified from control Nipponbare plant and from CRISPR/Cas9 mutant *hol1* $T_0$ lines 34.8, 41.3, and 57.

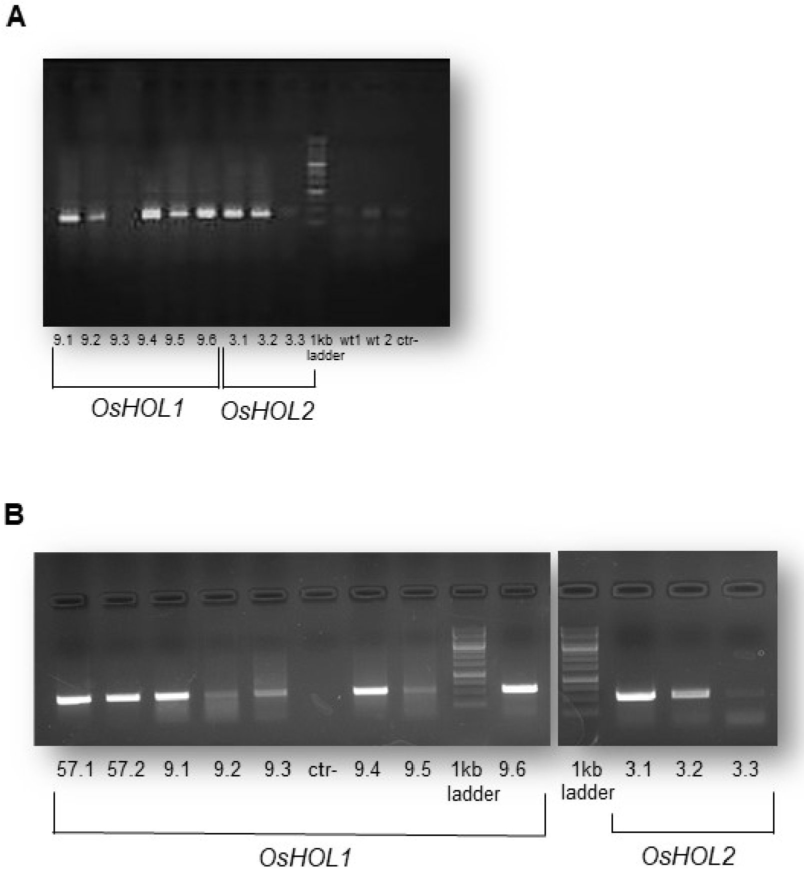


**Figure S5.** PCR analysis of $T_1$ CRISPR/Cas9 mutagenized lines. PCR amplification for the hygromycin resistance gene (**A**) and for the genomic region surrounding the gRNA target sequence in the gene of interest (**B**). Wild type (wt) samples (untransformed plant) and Ctr- sample (PCR negative control) were included in the analyses. Only the results of some $T_1$ plants carrying the construct for the mutagenesis of *OsHOL1* or *OsHOL2* are shown. The number of the lines analyzed are reported under the corresponding lane position in the gels. The GeneRuler™ 1kb DNA ladders (Thermo Scientific) are shown in the gels. Original full-length gels are presented in Figure S9.

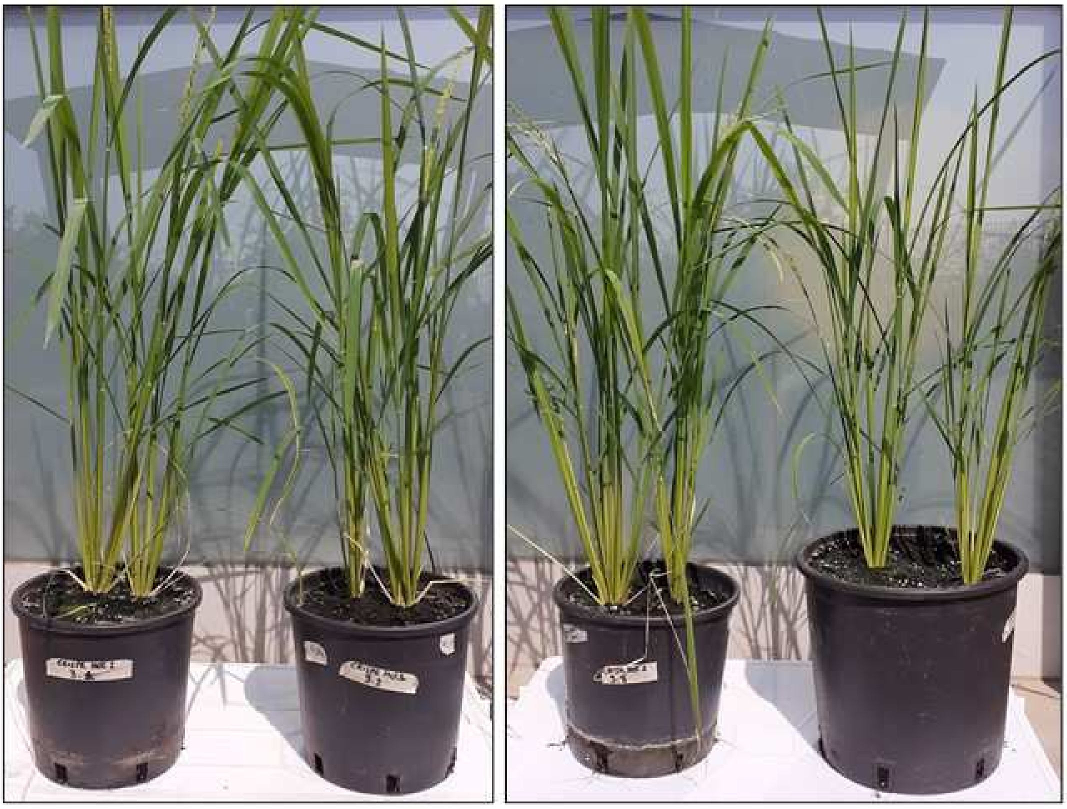


**Figure S6.** $T_1$ CRISPR/Cas9 *hol1* and *hol2* representative mutant plants compared to Nipponbare control plant. From left to right: *hol2*-L3.1, *hol1*-L9.2, *hol2*-L3.3 and control plants.

```
Os03g62670.1     ATGGCGTCGGCGATCGTCGACGTCGCCGGAGGCGGCCGGCAGCAAGCCCTCGACGGCTCG
OsHOL1 amplicon  ATGGCGTCGGCGATCGTCGACGTCGCCGGAGGCGGCCGGCAGCAAGCCCTCGACGGCTCG
                 ************************************************************

Os03g62670.1     AACCCGGCGGTGGCACGGCTGCGTCAGCTCATCGGCGGCGGCCAAGAATCGTCGGATGGG
OsHOL1 amplicon  AACCCGGCGGTGGCACGGCTGCGTCAGCTCATCGGCGGCGGCCAAGAATCGTCGGATGGG
                 ************************************************************

Os03g62670.1     TGGAGCAGGTGCTGGGAGGAAGGCGTGACGCCGTGGGATCTCGGCCAGCGGACGCCGGCC
OsHOL1 amplicon  TGGAGCAGGTGCTGGGAGGAAGGCGTGACGCCGTGGGATCTCGGCCAGCGGACGCCGGCC
                 ************************************************************

Os03g62670.1     GTTGTCGAGCTGGTGCACTCCGGGACCCTCCCCGCCGGCGACGCCACCACCGTCCTCGTC
OsHOL1 amplicon  GTTGTCGAGCTGGTGCACTCCGGGACCCTCCCCGCCGGCGACGCCACCACCGTCCTCGTC
                 ************************************************************

Os03g62670.1     CCCGGCTGCGGCGCCGGATACGATGTGGTTGCACTGTCCGGCCCCGGCCGCTTCGTCGTC
OsHOL1 amplicon  CCCGGCTGCGGCGCCGGATACGATGTGGTTGCACTGTCCGGCCCCGGCCGCTTCGTCGTC
                 ************************************************************

Os03g62670.1     GGCCTCGATATCTGTGACACGGCCATCCAGAAGGCGAAGCAGCTGTCGGCGGCGGCGGCG
OsHOL1 amplicon  GGCCTCGATATCTGTGACACGGCCATCCAGAAGGCGAAGCAGCTGTCGGCGGCGGCGGCG
                 ************************************************************

Os03g62670.1     GCGGCGGCTGACGGCGGCGACGGGAGCAGCAGCTTCTTCGCCTTCGTCGCCGACGATTTC
OsHOL1 amplicon  GCGGCGGCTGACGGCGGCGACGGGAGCAGCAGCTTCTTCGCCTTCGTCGCCGACGATTTC
                 ************************************************************

Os03g62670.1     TTCACGTGGGAGCCGCCGGAGCCGTTCCATCTCATCTTCGACTACACATTCTTCTGTGCT
OsHOL1 amplicon  TTCACGTGGGAGCCGCCGGAGCCGTTCCATCTCATCTTCGACTACACATTCTTCTGTGCT
                 ************************************************************

Os03g62670.1     CTGCATCCGTCGATGAGGCCAGCATGGGCGAAGAGAATGGCCGACCTGCTACGACCGGAC
OsHOL1 amplicon  CTGCATCCGTCGATGAGGCCAGCATGGGCGAAGAGAATGGCCGACCTGCTACGACCGGAC
                 ************************************************************

Os03g62670.1     GGAGAGCTCATCACCCTCATGTATTTGGCTGAAGGACAAGAGGCCGGGCCACCATTCAAT
OsHOL1 amplicon  GGAGAGCTCATCACCCTCATGTATTTGGCTGAAGGACAAGAGGCCGGGCCACCATTCAAT
                 ************************************************************

Os03g62670.1     ACAACAGTGCTCGATTACAAGGAGGTGCTGAACCCGTTGGGTTTAGTCATTACTTCTATC
OsHOL1 amplicon  ACAACAGTGCTCGATTACAAGGAGGTGCTGAACCCGTTGGGTTTAGTCATTACTTCTATC
                 ************************************************************

Os03g62670.1     GAGGACAATGAAGTCGCAGTTGAACCACGAAAGGGGATGGAGAAAATCGCAAGGTGGAAG
OsHOL1 amplicon  GAGGACAATGAAGTCGCAGTTGAACCACGAAAGGGGATGGAGAAAATCGCAAGGTGGAAG
                 ************************************************************

Os03g62670.1     AGGATGACAAAATCCGACTAA
OsHOL1 amplicon  AGGATGACAAAATCCGACTAA
                 *********************
```

**Figure S7.** ClustalW alignment of *OsHOL1* transcripts. The *OsHOL1* longest predicted transcript (*Os03g62670.1*) and the cds cloned in the study from *OsHOL1* are shown. CLUSTAL W Version 1.83 was used (https://www.genome.jp/tools-bin/clustalw).

**A**

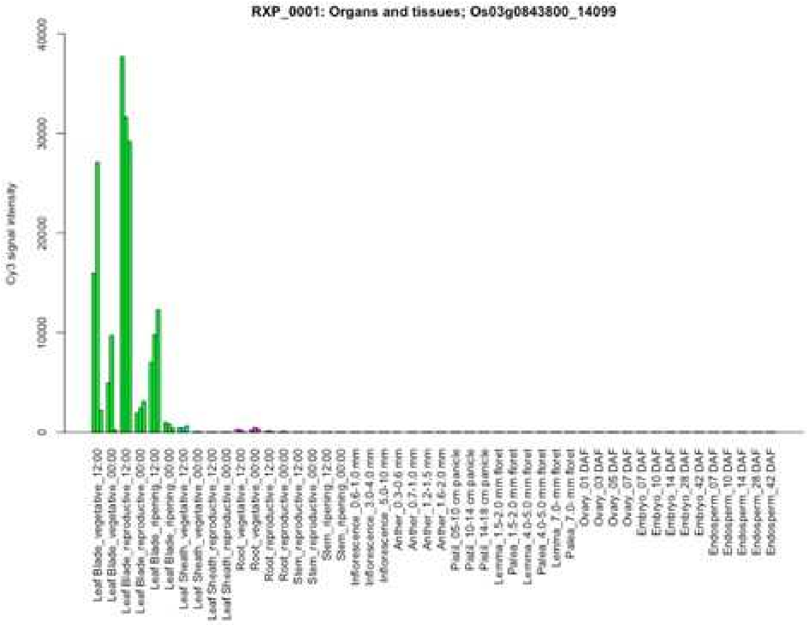


**B**

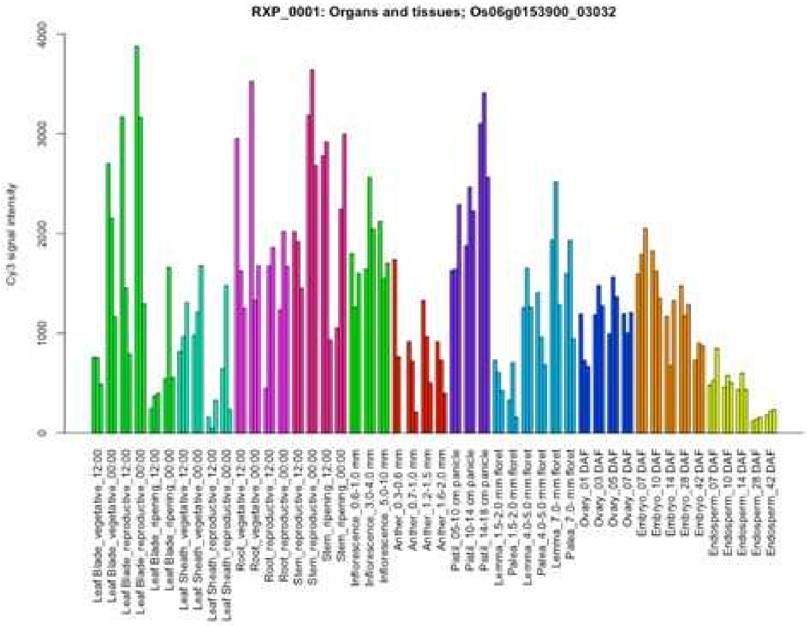


**Figure S8.** Outputs of rice expression profile in organs and tissues for *OsHOL1* (Os03g0843800) and *OsHOL2* (Os06g0153900). Expression profiles are from RiceXPro (RXP) (http://ricexpro.dna.affrc.go.jp).

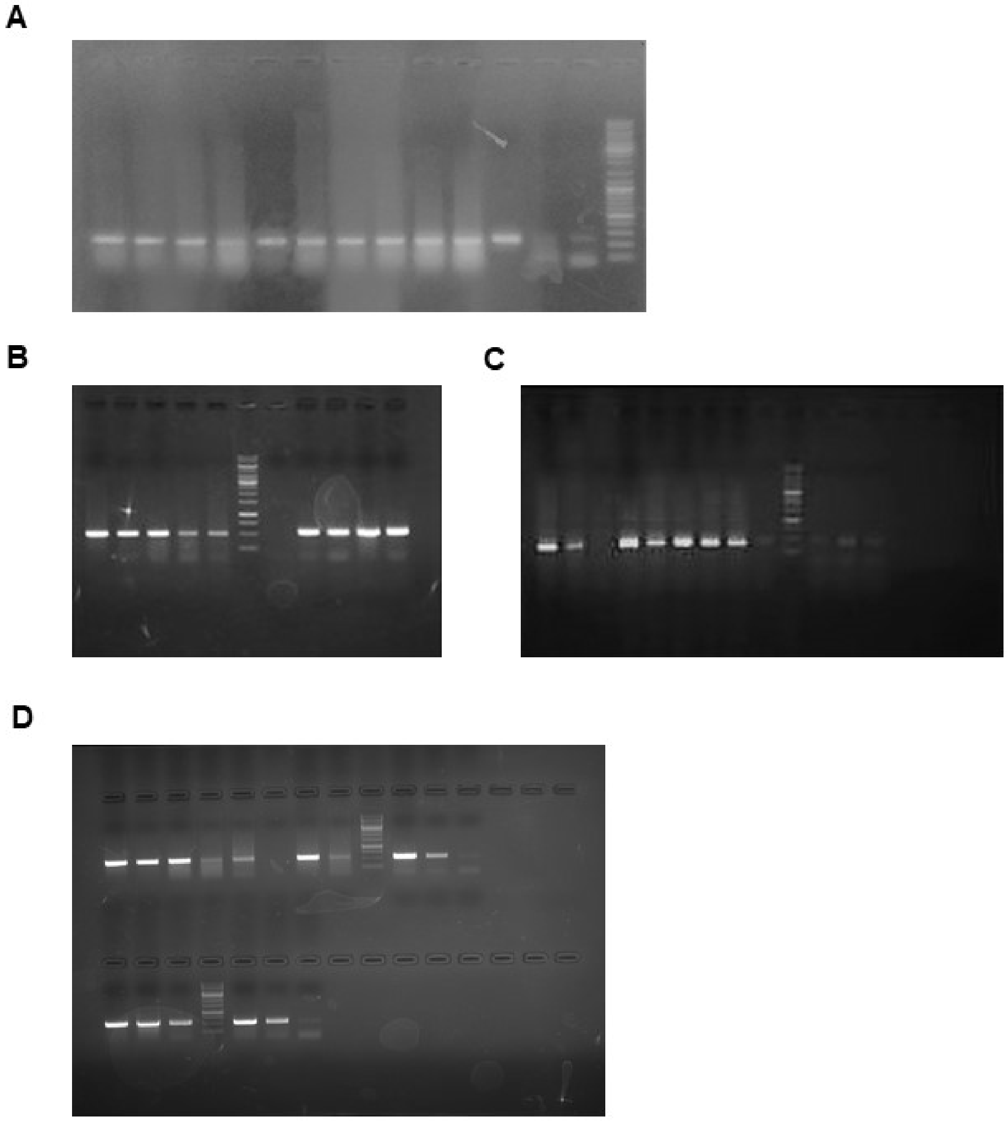

**Figure S9.** Original images of the electrophoretic gels shown in Figure S3 (**A, B**) and Figure S4 (**C**, **D**). The photos of the gels A, B, and C are the same shown in Figure S3A, B and S4A, without any adjustments. The picture D corresponds to the photo of the uncropped electrophoretic gel from which the two small gels shown in Figure S4B were taken, without any adjustment. All the images were acquired with the "ChemiDoc Imaging System" (Bio-Rad).

| TARGET GENE | MUTANT LINE | MUTATION TYPE | TARGET GENE | MUTANT LINE | MUTATION TYPE |
|---|---|---|---|---|---|
| *OsHOL1* | 1 | +1/+1 | *OsHOL2* | 2 | -1/-1 |
| *OsHOL1* | 1.1 | +1/-6/-6 | *OsHOL2* | 3 | +1/+1 |
| *OsHOL1* | 1.2 | +1/-6/-6/-6 | *OsHOL2* | 5 | +1/+1 |
| *OsHOL1* | 1.3 | -20/-24/-24/-20 | *OsHOL2* | 10.3 | +2/-1 |
| *OsHOL1* | 1.5 | +3/-5/-8 | *OsHOL2* | 10.4 | +2/-1 |
| *OsHOL1* | 1.10 | +1/+1 | *OsHOL2* | 11.2 | -2/0 |
| *OsHOL1* | 9 | +1/+1 | *OsHOL2* | 11.3 | +1/-1 |
| *OsHOL1* | 20.1 | +1/-1/-17/-17/-17 | *OsHOL2* | 11.8 | +1/-7/-7 |
| *OsHOL1* | 21.1 | +1/-6/-6 | *OsHOL2* | 13 | -2/-2 |
| *OsHOL1* | 22.2 | +1/+1 | *OsHOL2* | 13.1 | -1/-3/-3 |
| *OsHOL1* | 24.1 | +1/+1 | *OsHOL2* | 15 | -1/-8/-8 |
| *OsHOL1* | 24.3 | -4/-4/-4/-4 | *OsHOL2* | 16.1 | -4/-4 |
| *OsHOL1* | 25.6 | +1/-6/-6 | *OsHOL2* | 16.2 | -4/-4 |
| *OsHOL1* | 25.10 | +1/-3/-3 | *OsHOL2* | 18 | +1/-17/-17/-17 |
| *OsHOL1* | 27.10 | +1/-6/-6/0/-12 | *OsHOL2* | 20.1 | +1/-17/-17/-17 |
| *OsHOL1* | 29 | +1/ +1 | *OsHOL2* | 50.1 | -4/-4 |
| *OsHOL1* | 29.1 | +1/-6/-6 | *OsHOL2* | 50.2 | -4/-4 |
| *OsHOL1* | 31 | +1/-1 | *OsHOL2* | 60.9 | -29/-2 |
| *OsHOL1* | 34.1 | +1/-1 | *OsHOL2* | 60.11 | -1/-7/-7 |
| *OsHOL1* | 34.8 | +1/+1 | *OsHOL2* | 60.12 | -1/-7/-7 |
| *OsHOL1* | 35 | +1/+1 | | | |
| *OsHOL1* | 35.1 | -6/-8/-8 | | | |
| *OsHOL1* | 35.3 | +1/+1 | | | |
| *OsHOL1* | 37.1 | -2/-2 | | | |
| *OsHOL1* | 38 | +1/+1 | | | |
| *OsHOL1* | 38.1 | +21/+1 | | | |
| *OsHOL1* | 38.2 | +2/-12/+1/-4 | | | |
| *OsHOL1* | 40 | +1/+1 | | | |
| *OsHOL1* | 41.1 | -26/-21 | | | |
| *OsHOL1* | 41.3 | -26/-21 | | | |
| *OsHOL1* | 42 | +1/-2 | | | |
| *OsHOL1* | 46.2 | +3/-5/-8 | | | |
| *OsHOL1* | 53 | -3/-2/-3/-2/-3 | | | |
| *OsHOL1* | 57 | +1/0 | | | |

**Table S1.** List of the mutations generated in *OsHOL1* and *OsHOL2* in the different $T_0$ lines. Mutation type indicates the number and types of indels identified in the gene of interest. When more than two indels were found, the genotype of the mutant line was chimeric. Indels are ordered according to their relative frequencies (not reported).

| GENE | FORWARD primer | REVERSE primer |
|---|---|---|
| *OsGAPDH (RT-qPCR)* | AAGCCAGCATCCTATGATCAGATT | CGTAACCCAGAATACCCTTGAGTTT |
| *OsHOL1 (RT-qPCR)* | GCCGTTCCATCTCATCTTCGA | CCAAATACATGAGGGTGATGAGC |
| *OsHOL2 (RT-qPCR)* | TGGCTGGGAGAAGTCGTG | GAGAGTTCCTGATTTAACGAGATGT |
| *OsHOL1* - *sg*RNA | GGCACTCCCACGTGAAGAAATCGT | AAACACGATTTCTTCACGTGGGAG |
| *OsHOL2* - *sg*RNA | GGCAGTTGGCTTCCCCAAATCCCA | AAACTGGGATTTGGGGAAGCCAAC |
| *hygromycin (hph)* | TGCATCGAAATTGCCGT | CGATTGCTGATCCCCATGTG |
| *OsHOL1 - target sequence* | CAAATAGGGTAACAAAATGCATGA | ACTCGAGCACTGTTGTATTGAATG |
| *OsHOL2 - target sequence* | GAGGATGGATCGGGCTCTC | TAACGAGATGTTCGATGATAGGTG |
| *OsHOL2 - off-target sequence* | GAAGTATCTAATCCTTTTATCCGTTGGT | TCTTCAGAAGGATAGGCTATGAATACAA |
| *OsHOL1 cds* | CACCATGGCGTCGGCGATCGTC | TTAGTCGGATTTTGTCATCCTCTTC |

**Table S2.** List of the oligonucleotide primers used in the PCR reactions.
